# Supplementary material for: Clinical associations with a polygenic predisposition to benign lower white blood cell counts
Source: Nat Commun. 2024 Apr 22;15:3384. doi: 10.1038/s41467-024-47804-5 (PMC11035609; doi:10.1038/s41467-024-47804-5)
Supplement: Supplementary file 1 — Supplementary Information [file 41467_2024_47804_MOESM1_ESM.pdf]

Supplemental Figure and Tables

1. **Supplemental Figure 1.** Associations with hematological malignancies and auto-immune diseases.

2. **Supplemental Figure 2:** PGS associations with measured minimum WBC counts among 11,694 BioVU participants.

3. **Supplemental Figure 3:** Hosmer-Lemeshow goodness-of fit plot from the logistic regression model between the PGS_{WBC} and having an ICD code related to a low WBC count.

4. **Supplemental Figure 4:** Kaplan-Meier plot of frequency of neutropenia with taxane treatment.

5. **Supplemental Figure 5:** Associations with drug induced leukopenia due to azathioprine in the Michigan Genomics Initiative cohort.

6. **Supplemental Figure 6:** Scatter plots of the first 2 genetic principal components for the HapMap reference populations and the BioVU cohort.

7. **Supplemental Figure 7:** Distribution of the PGS_{WBC} in the BioVU cohort and the individual study populations.

8. **Supplemental Table 1:** Associations between the PGS_{WBC} and malignant hematological malignancies and auto-immune diseases.

9. **Supplemental Table 2:** Association between the PGS_{WBC} based on quintiles of SNPs constructed based on the distribution of SNP effect sizes and malignant hematological phenotypes.

10. **Supplemental Table 3:** Count of the number of malignant hematological phenotypes with an excess of nominal SNP associations, stratified by quintiles based on the distribution of SNP effect sizes.

11. **Supplemental Table 4:** Sensitivity analyses examining the impacts of excluding SNPs from the PGS_{WBC} that are nominally associated with hematological phenotypes at select significance thresholds.

12. **Supplemental Table 5:** Characteristics of BioVU participants in the bone marrow biopsy cohort.

13. **Supplemental Table 6:** Sensitivity analyses examining the impacts of excluding SNPs from the PGS_{WBC} that are nominally associated with hematological phenotypes at select significance thresholds on biopsy outcomes.

14. **Supplemental Table 7:** Characteristics of BioVU participants in the taxane leukopenia study.

15. **Supplemental Table 8:** Characteristics of Michigan Genomics Initiative participants in the azathioprine leukopenia study.

16. **Supplemental Table 9:** Characteristics of BioVU participants in the azathioprine discontinuation study.

17. **Supplemental Table 10:** List of keywords used to identify records with a mention of a low WBC count.

18. **Supplemental Table 11:** Diseases with associations reported in the GWAS catalog for which SNPs were excluded when constructing the PGS_{WBC}.

Supplemental Figure 1. Associations with hematological malignancies and auto-immune diseases. Associations between the PGS_{WBC} and (A) hematological malignancies and (B) auto-immune diseases present among 71,078 BioVU participants. Odds-ratios are per standard deviation change in the PGS_{WBC}. For reference, associations with diagnoses of a decreased WBC count and neutropenia are shown in the same population.

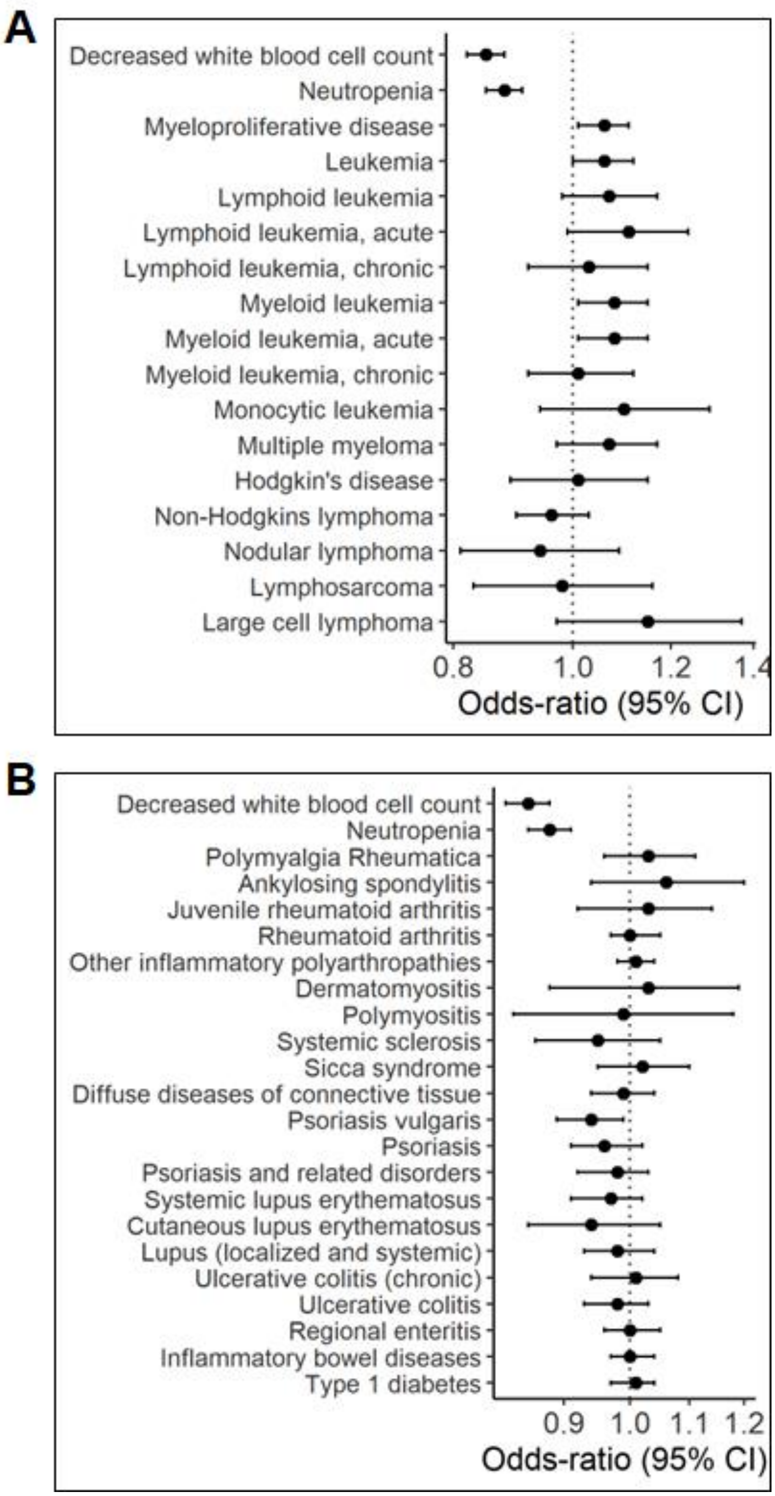

Supplemental Figure 2: PGS associations with measured minimum WBC counts among 11,694 BioVU participants. (**a**) Ranges of observed minimum WBC counts by PGS_{WBC} value. Ranges summarize WBC counts within sequential windows (+/- 0.2 s.d.) across the range of the PGS_{WBC}. The dark line is the median value, the light grey ribbon represents the 5th to 95th percentiles of the range, and the dark grey ribbon represents the 2.5th to 5th percentile. The dashed red lines denote the upper and lower clinical reference ranges for the clinical assay used to measure the WBC count.

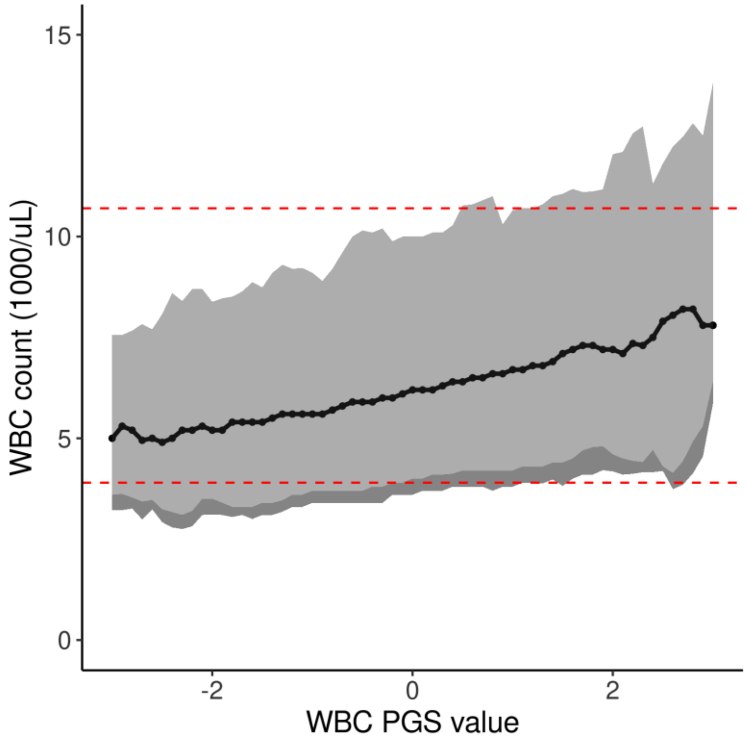

Supplemental Figure 3: Hosmer-Lemeshow goodness-of fit plot from the logistic regression model between the PGS_{WBC} and having an ICD code related to a low WBC count.

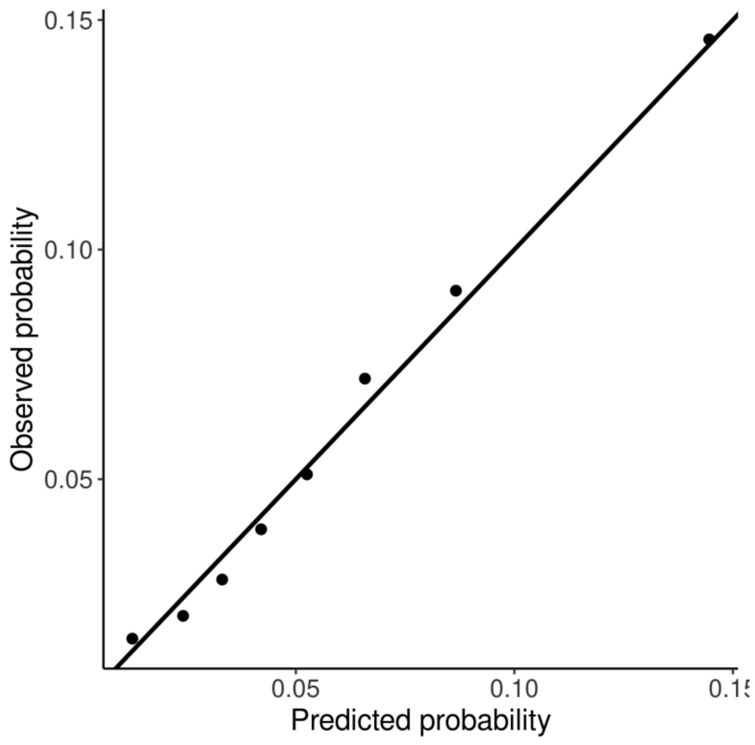

Supplemental Figure 4: Kaplan-Meier plot of frequency of neutropenia with taxane treatment. Kaplan-Meier curves summarizing frequencies of neutropenia (absolute neutrophil count<1,500 cells/µl) after initiating treatment with taxane medications among 1,724 BioVU participants with a primary cancer. The low and high PGS_{WBC} groups correspond to values <1 s.d. below the mean and >1 s.d. above the mean (high), respectively. The y-axis is the proportion of individuals with incident neutropenia.

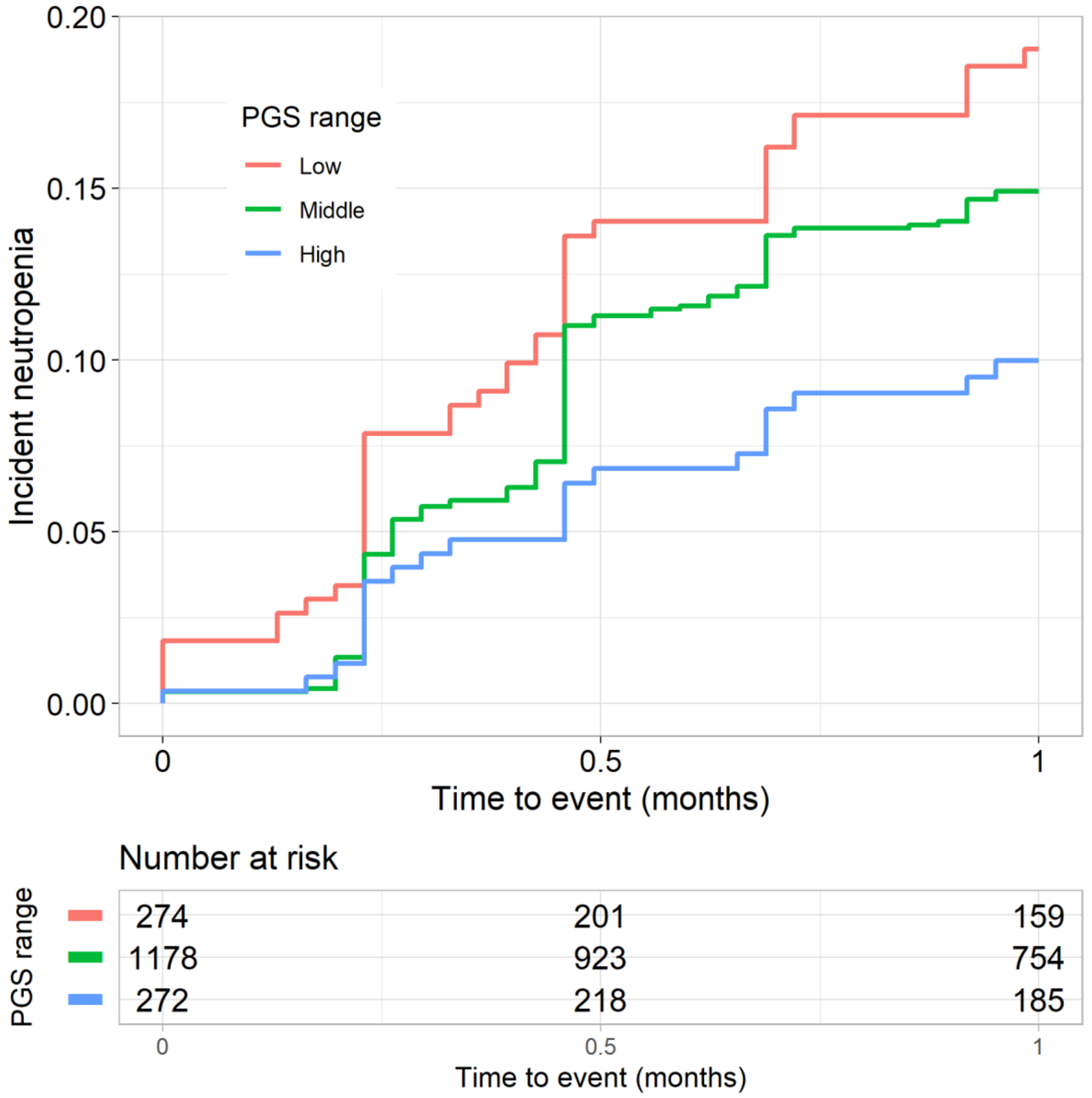

Supplemental Figure 5: Associations with drug induced leukopenia due to azathioprine in the Michigan Genomics Initiative cohort. (A) Summary of associations between the PGS and incident leukopenia, defined at different WBC count thresholds. (B) Kaplan-Meier plot of frequency of leukopenia with azathioprine treatment. Kaplan-Meier curves summarizing frequencies of a WBC count<4,000 cells/ul after initiating treatment with azathioprine among 354 MGI participants with auto-immune disease. The low and high PGS groups correspond to values <1 s.d. below the mean and >1 s.d. above the mean (high), respectively.

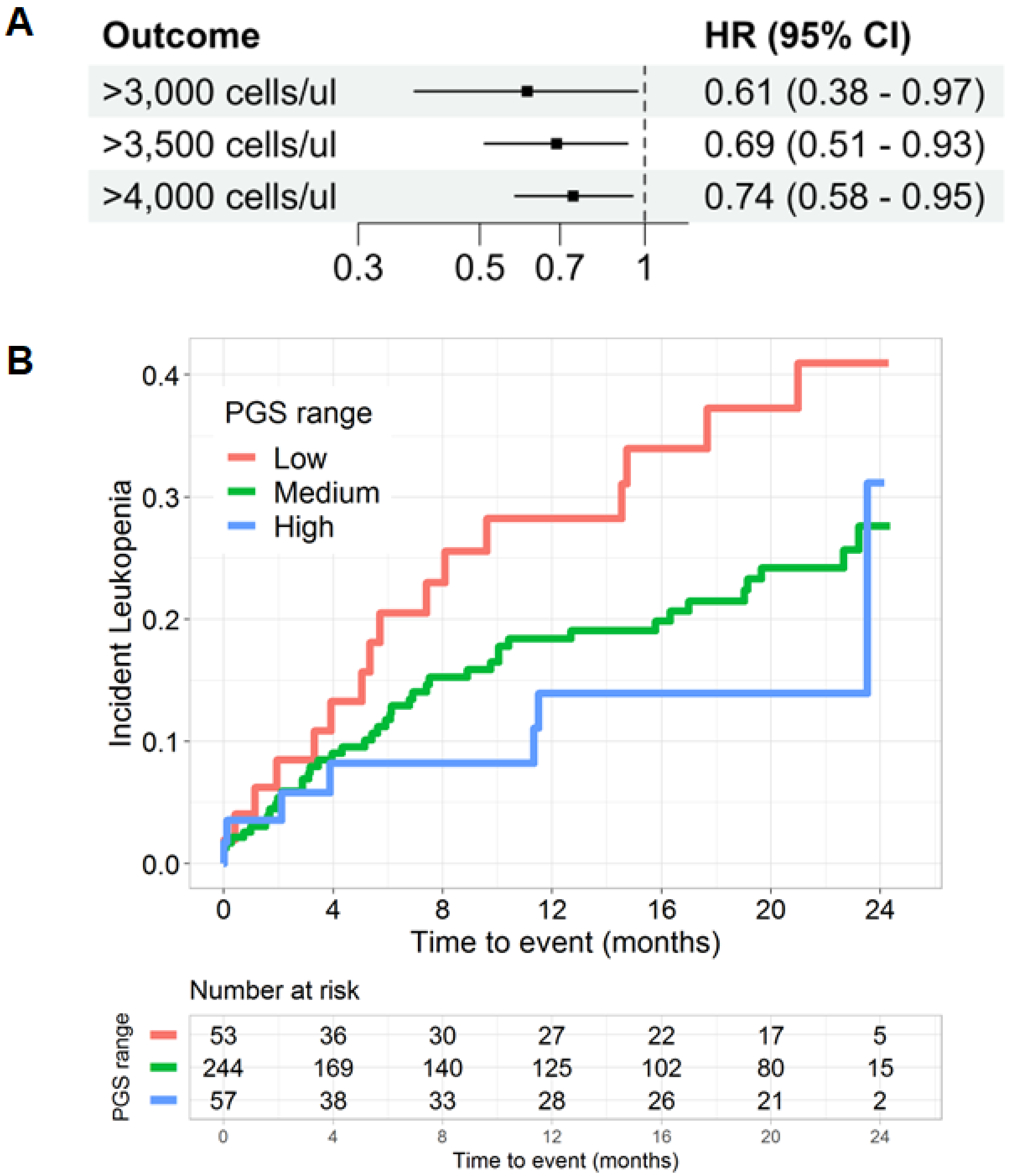

Supplemental Figure 6: Scatter plots of the first 2 genetic principal components for the HapMap reference populations and the BioVU cohort. Principal components were fit to the combined BioVU and HapMap data sets. A) HapMap reference populations. B) The BioVU population. Participants identified as EA are included in the present study and are referred to as "European ancestry".

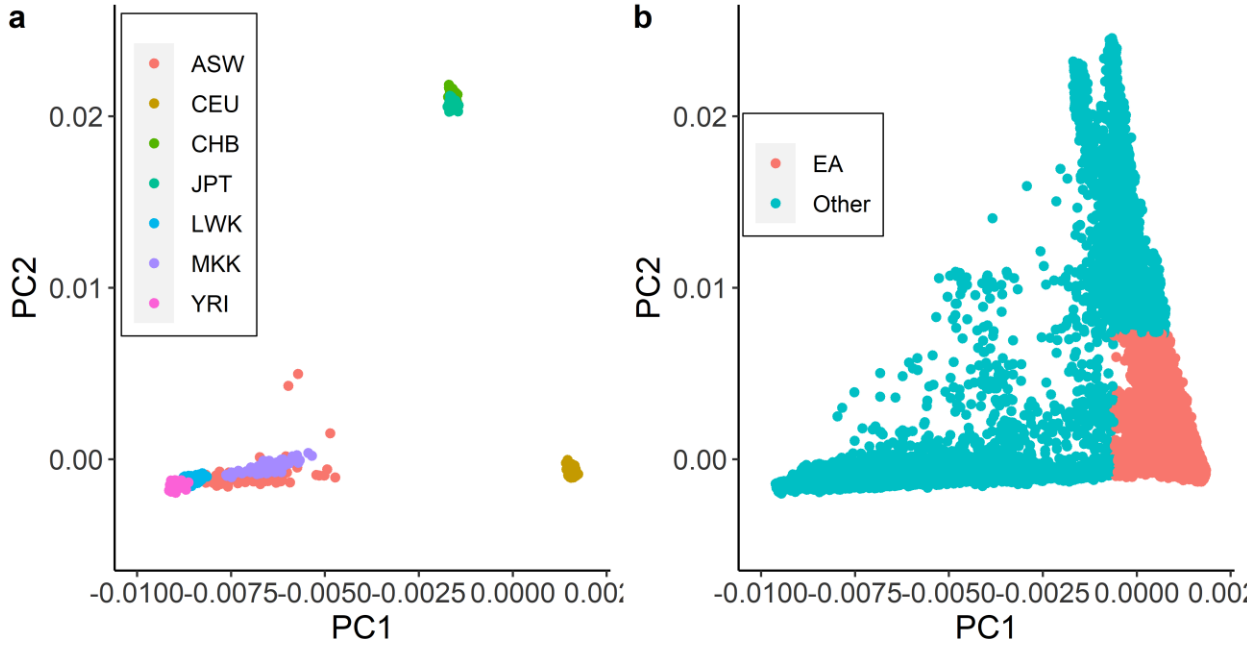

Supplemental Figure 7: Distribution of the PGS_{WBC} in the BioVU cohort and the individual study populations. The PGS was set to have mean of 0 and standard deviation of 1 in the full BioVU European ancestry population ("All", n= 71,078)

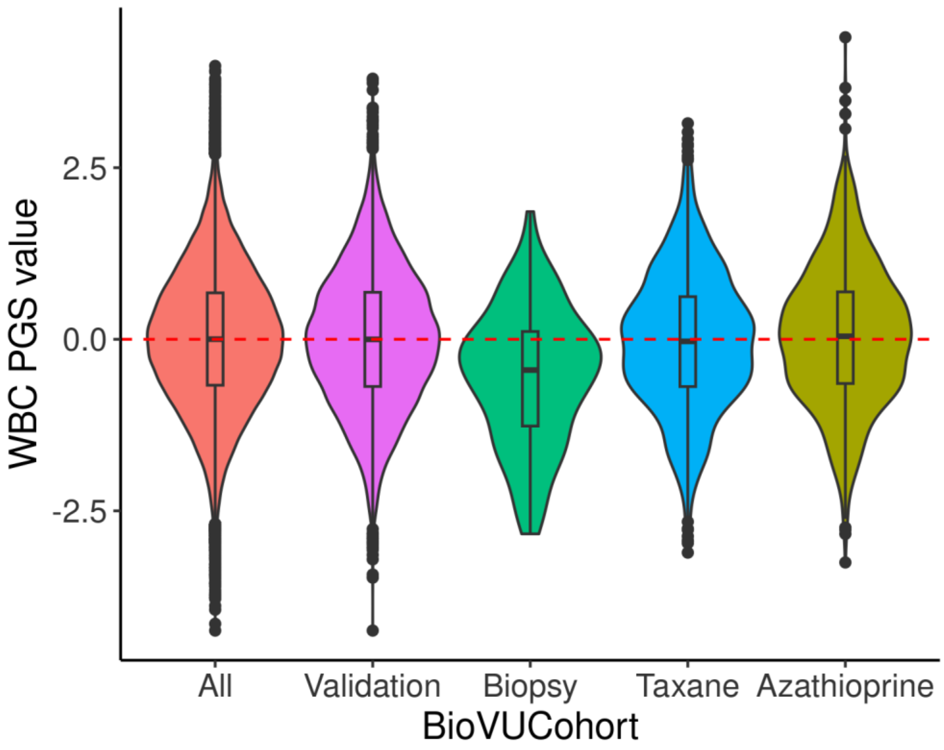

Supplemental Table 1: Associations between the PGS_{WBC} and PheWAS codes for auto-immune diseases and malignant hematological malignancies among BioVU participants. Odds ratios are from a logistic regression model, adjusted for age, sex and PCs. P-values are based on a 2-sided test. False Discovery Rate p-values are presented.

PheWAS Code	Description	Number of cases	Number of controls	Odds ratio	95% CI	p-value	FDR p-value
200	Myeloproliferative disease	1778	66075	1.06	(1.01 - 1.11)	0.03	0.18
201	Hodgkin's disease	251	64757	1.01	(0.89 - 1.15)	0.88	0.90
202.2	Non-Hodgkins lymphoma	905	65268	0.96	(0.9 - 1.03)	0.22	0.63
202.21	Nodular lymphoma	189	64674	0.94	(0.81 - 1.09)	0.41	0.71
202.23	Lymphosarcoma	150	64682	0.98	(0.83 - 1.16)	0.83	0.90
202.24	Large cell lymphoma	140	64565	1.15	(0.97 - 1.37)	0.10	0.42
204	Leukemia	1500	65847	1.06	(1.00 - 1.12)	0.03	0.18
204.1	Lymphoid leukemia	528	65843	1.07	(0.98 - 1.17)	0.12	0.44
204.11	Lymphoid leukemia, acute	321	65632	1.11	(0.99 - 1.24)	0.07	0.35
204.12	Lymphoid leukemia, chronic	336	65573	1.03	(0.92 - 1.15)	0.59	0.84
204.2	Myeloid leukemia	945	65631	1.08	(1.01 - 1.15)	0.02	0.18
204.21	Myeloid leukemia, acute	881	65665	1.08	(1.01 - 1.15)	0.03	0.18
204.22	Myeloid leukemia, chronic	436	60491	1.01	(0.92 - 1.12)	0.76	0.90
204.3	Monocytic leukemia	163	62031	1.10	(0.94 - 1.29)	0.24	0.63
204.4	Multiple myeloma	461	57930	1.07	(0.97 - 1.17)	0.17	0.52
250.1	Type 1 diabetes	3697	47540	1.01	(0.97 - 1.04)	0.64	0.84
555	Inflammatory bowel disease and other gastroenteritis and colitis	2935	43248	1.00	(0.97 - 1.04)	0.82	0.90
555.1	Regional enteritis	2489	43328	1.00	(0.96 - 1.05)	0.85	0.90
555.2	Ulcerative colitis	1907	43022	0.98	(0.93 - 1.03)	0.37	0.70
555.21	Ulcerative colitis (chronic)	826	43110	1.01	(0.94 - 1.08)	0.86	0.90
695.4	Lupus (localized and systemic)	1222	54717	0.98	(0.93 - 1.04)	0.61	0.84
695.41	Cutaneous lupus erythematosus	345	55276	0.94	(0.85 - 1.05)	0.28	0.63
695.42	Systemic lupus erythematosus	1264	54603	0.97	(0.91 - 1.02)	0.26	0.63
696	Psoriasis and related disorders	1405	55312	0.98	(0.92 - 1.03)	0.39	0.70
696.4	Psoriasis	1270	54084	0.96	(0.91 - 1.02)	0.16	0.52
696.41	Psoriasis vulgaris	1384	54013	0.94	(0.89 - 0.99)	0.03	0.18
709	Diffuse diseases of connective tissue	1664	56440	0.99	(0.94 - 1.04)	0.64	0.84
709.2	Sicca syndrome	738	51237	1.02	(0.95 - 1.10)	0.52	0.84
709.3	Systemic sclerosis	405	55454	0.95	(0.86 - 1.05)	0.28	0.63
709.4	Polymyositis	130	56448	0.99	(0.83 - 1.18)	0.92	0.92
709.5	Dermatomyositis	178	57064	1.03	(0.88 - 1.19)	0.72	0.90
714	Rheumatoid arthritis and other inflammatory polyarthropathies	3577	58856	1.01	(0.98 - 1.04)	0.59	0.84
714.1	Rheumatoid arthritis	2623	58957	1.00	(0.97 - 1.05)	0.81	0.90
714.2	Juvenile rheumatoid arthritis	382	56050	1.03	(0.92 - 1.14)	0.63	0.84
715.2	Ankylosing spondylitis	268	53095	1.06	(0.94 - 1.20)	0.33	0.69
717	Polymyalgia Rheumatica	738	67673	1.03	(0.96 - 1.11)	0.39	0.70

Supplemental Table 2: Association between WBC PGSs constructed based on quintiles of the distribution of SNP effect sizes and malignant hematological phenotypes.

SNP effect size quintile[1]	Number of associated hematological phenotypes[2]	Probability[3]	Number of positive associations[4]	Number of negative associations[4]
1	0	1	0	0
2	3	0.129	0	3
3	1	0.464	0	1
4	8	0.001	8	0
5	1	0.466	1	0

Notes:

1. The lowest quintiles represent the SNPs with the smallest effect sizes.
2. Number of phenotypes with a nominal ($p<0.05$) association with the quintile-PGS among 15 malignant hematological phenotypes evaluated.
3. Probability of an association count greater than equal to that observed based on 1000 permutations of the PGS.
4. Number of the associations that are in a positive or negative (inverse) direction.

Supplemental Table 3: Count of the number of malignant hematological phenotypes with an excess of nominal SNP associations, stratified by quintiles based on the distribution of SNP effect sizes.

SNP effect size quintile[1]	Number of hematological phenotypes with excess SNP associations[2]
1	1
2	1
3	0
4	4
5	1

Notes:

1. The lowest quintiles represent the SNPs with the smallest effect sizes.
2. Number of phenotypes that had more nominal ($p<0.05$) SNP associations than expected (based on a binomial probability $p<0.05$) among 15 malignant hematological phenotypes evaluated.

Supplemental Table 4: Sensitivity analyses examining the impacts of excluding SNPs from the PGS_{WBC} that are nominally associated with hematological phenotypes at select significance thresholds. Odds ratios are from a logistic regression model, adjusted for age, sex and principal components. P-values are based on a 2-sided test.

**Association with having a WBC count outside the reference range.**				
**P-value threshold[1]**	**# SNPs excluded**	**Odds-ratio**	**95% CI**	**P-value**
0.001	33	0.58	(0.53 - 0.63)	1.9×10^{-38}
0.005	124	0.58	(0.53 - 0.63)	3.3×10^{-37}
0.01	223	0.58	(0.53 - 0.63)	4.2×10^{-36}
0.05	753	0.60	(0.55 - 0.65)	4.8×10^{-31}
**Association with ICD code for a low WBC count**				
**P-value threshold[1]**	**# SNPs excluded**	**Odds-ratio**	**95% CI**	**P-value**
0.001	33	0.63	(0.57 - 0.70)	2.6×10^{-18}
0.005	124	0.63	(0.57 - 0.70)	1.0×10^{-17}
0.01	223	0.63	(0.57 - 0.70)	1.5×10^{-17}
0.05	753	0.66	(0.59 - 0.74)	9.7×10^{-14}

Notes:

1. Association p-value used to exclude SNPs based on association testing between the PGS_{WBC} and 15 malignant hematological phenotypes.

Supplemental Table 5: Characteristics of BioVU participants in the bone marrow biopsy cohort.

Characteristic	All participants	Participants with a normal biopsy	Participants with an abnormal biopsy
n	117	82	35
Age (years) [mean (s.d.)]	48.4 (24.5)	44.4 (24.9)	58.0 (21.0)
Males (n [%])	53 (45.3%)	34 (41.5%)	19 (54.3%)
Age<18 years (n [%])	19 (16.2%)	16 (19.5%)	3 (8.6%)
**Indication for biopsy (n [%]):**			
Low WBC count	76 (65%)	53 (64.6%)	23 (65.7%)
Neutropenia (n [%])	51 (43.6%)	37 (45.1%)	14 (40%)
**Listed comorbidities (n [%]):**			
Any hematological diagnosis	66 (56.4%)	37 (45.1%)	29 (82.9%)
Thrombocytopenia	30 (25.6%)	19 (23.2%)	11 (31.4%)
Anemia	40 (34.2%)	22 (26.8%)	18 (51.4%)
Other hematological issue	13 (11.1%)	7 (8.5%)	6 (17.1%)
Other non-hematological diagnosis	13 (11.1%)	9 (11%)	4 (11.4%)
**Biopsy results (n [%]):**			
Normal biopsy (no pathology)	82 (70.1%)	82 (100%)	0 (0%)
Any WBC-related abnormality	28 (23.9%)	0 (0%)	28 (80%)

Supplemental Table 6: Sensitivity analyses examining the impacts of excluding SNPs from the PGS_{WBC} that are nominally associated with hematological phenotypes at select significance thresholds on biopsy outcomes. Odds ratios are from a logistic regression model, adjusted for age, sex and principal components. P-values are based on a 2-sided test.

P-value threshold[1]	# SNPs excluded	Odds-ratio[2]	95% CI	P-value
0.001	33	0.59	(0.34 - 0.97)	0.048
0.005	124	0.57	(0.32 - 0.96)	0.04
0.01	223	0.54	(0.30 - 0.94)	0.04
0.05	753	0.49	(0.26 - 0.85)	0.02

Notes:

1. Association p-value used to exclude SNPs based on association testing between the PGS_{WBC} and 15 malignant hematological phenotypes.
2. Odds-ratio for a having a biopsy outcome that does not identify disease.

Supplemental Table 7: Characteristics of BioVU participants in the taxane leukopenia study.

Characteristic	All participants
n	1724
Females (n [%])	917 (53.2%)
Age at treatment (mean [s.d.])	59.5 (12)
Baseline WBC (x1000 cells/µl)	8.2 (3.9)
Baseline ANC (x1000 cells/µl)	5.8 (3.7)
Cancer diagnosis:	
Bone/connective tissues cancer	7 (0.4%)
Breast Cancer	383 (22.2%)
Hematopoietic	5 (0.3%)
Lung cancer	256 (14.8%)
Oral Cancer	301 (17.5%)
Respiratory cancer	92 (5.3%)
Genitourinary cancer	115 (6.7%)
Other cancers	565 (32.7%)
Drug treatment:	
Docetaxel (n [%])	406 (23.5%)
Paclitaxel (n [%])	1318 (76.5%)
Mean dose (mg/m^2)	80.5 (47)
Number treatments	7.1 (6.3)
Prior GCSF treatment (n [%])	419 (24.3%)
Outcomes:	
WBC<3000 cells/µl	266 (15.5%)
Follow-up time (months)	0.8 (0.3)
ANC<1500 cells/µl	228 (13.2%)
Follow-up time (months)	0.8 (0.3)

Supplemental Table 8: Characteristics of Michigan Genomics Initiative participants in the azathioprine leukopenia study.

Characteristic	All Subjects
n	354
Females	203 (57.3%)
Age at treatment (mean [s.d.])	43.5 (16.6)
Baseline WBC (x1000 cells/ul)	8.8 (3.7)
Final WBC (x1000 cells/ul)	7.3 (3.0)
Change in WBC (x1000 cells/ul)	1.5 (3.6)
Incident events (n, %):	
WBC count<3,000	17 (4.8%)
WBC count<3,500	45 (12.7%)
WBC count<4,000	67 (18.9%)
Follow-up duration (months)	18.2 (9.0)

Supplemental Table 9: Characteristics of participants in the azathioprine discontinuation study.

Characteristic	All participants	Participants discontinued	Participants not discontinued
n	1,180	34	1,146
Females (n [%])	787 (66.7%)	24 (70.6%)	763 (66.6%)
Age (years) [mean (s.d.)]	46.7 (15.2)	49.5 (18)	46.6 (15.1)
Underlying diagnosis (n [%]):			
Lupus	114 (9.7%)	3 (8.8%)	111 (9.7%)
Inflammatory bowel disease	418 (35.4%)	11 (32.4%)	407 (35.5%)
Other	648 (54.9%)	20 (58.8%)	628 (54.8%)
Baseline WBC (x1,000 cells/µl)	8.6 (3.4)	7 (3.7)	8.6 (3.4)
Final WBC (x1,000 cells/ul)	7.3 (3.4)	3.7 (1.7)	7.5 (3.4)
Change in WBC (x1,000 cells/µl)	1.4 (3.8)	3.9 (3.2)	1.3 (3.8)
Initial dose (mg)	80.7 (48.9)	77.2 (45.8)	80.8 (49)
Discontinued for low WBC or neutrophil counts (n [%])	34 (2.9%)	34 (100%)	0 (0%)
Follow-up duration (months) (mean [s.d])	14.4 (10)	7.3 (5.8)	14.6 (10.1)

Supplemental Table 10: List of keywords used to identify records with a mention of a low WBC count.

Keyword
leukopenia
leucopenia
low white
decreased white
reduced white
low wbc
decreased wbc
reduced wbc
neutropenia
low neutro
decreased neutro
reduced neutro
lymphopenia
anc
lymphopenia
granulopenia
agranulocytosis
nutropenia
neutropenai
nuetropenia

Supplemental Table 11: Diseases with associations reported in the GWAS catalog for which SNPs were excluded when constructing the WBC PGS.

Disease
Systemic lupus erythematosus
Chronic lymphocytic leukemia
Multiple myeloma
Myeloproliferative neoplasms
Hodgkin's lymphoma
Follicular lymphoma
Lymphoma
Nodular sclerosis Hodgkin lymphoma
Acute lymphoblastic leukemia in childhood (B cell precursor)
Lymphocytic leukemia
Marginal zone lymphoma
Acute lymphoblastic leukemia (B-cell precursor)
Acute lymphoblastic leukemia (childhood)
B-cell acute lymphoblastic leukaemia
B-cell malignancies (chronic lymphocytic leukemia, Hodgkin lymphoma or multiple myeloma)
Diffuse large B cell lymphoma
Extranodal natural killer T-cell lymphoma (nasal type)
Multiple myeloma (hyperdiploidy)
Multiple myeloma (IgH translocation)
Multiple myeloma and monoclonal gammopathy
Primary central nervous system lymphoma
Severe aplastic anemia
