# Supplementary material for: Clinical associations with a polygenic predisposition to benign lower white blood cell counts
Source: Nat Commun. 2024 Apr 22;15:3384. doi: 10.1038/s41467-024-47804-5 (PMC11035609; doi:10.1038/s41467-024-47804-5)
Supplement: Supplementary file 3 — Description of Additional Supplementary Files [file 41467_2024_47804_MOESM3_ESM.pdf]

Description of Additional Supplementary Files

File Name: Supplementary Data 1
Description: ICD-9 and ICD-10 codes related to chemotherapies and hematological diagnoses.

File Name: Supplementary Data 2
Description: SNPs and weightings retained in the WBC PGS.
