# Supplementary material for: Clinical associations with a polygenic predisposition to benign lower white blood cell counts
Source: Nat Commun. 2024 Apr 22;15:3384. doi: 10.1038/s41467-024-47804-5 (PMC11035609; doi:10.1038/s41467-024-47804-5)
Supplement: Supplementary file 5 — Reporting Summary [file 41467_2024_47804_MOESM5_ESM.pdf]

Corresponding author(s): Jonathan Mosley

Last updated by author(s): 2024-93-13

Reporting Summary

Nature Portfolio wishes to improve the reproducibility of the work that we publish. This form provides structure for consistency and transparency in reporting. For further information on Nature Portfolio policies, see our Editorial Policies and the Editorial Policy Checklist.

Statistics

For all statistical analyses, confirm that the following items are present in the figure legend, table legend, main text, or Methods section.

n/a	Confirmed	
☐	☒	The exact sample size (*n*) for each experimental group/condition, given as a discrete number and unit of measurement
☐	☒	A statement on whether measurements were taken from distinct samples or whether the same sample was measured repeatedly
☐	☒	The statistical test(s) used AND whether they are one- or two-sided<br>*Only common tests should be described solely by name; describe more complex techniques in the Methods section.*
☐	☒	A description of all covariates tested
☐	☒	A description of any assumptions or corrections, such as tests of normality and adjustment for multiple comparisons
☐	☒	A full description of the statistical parameters including central tendency (e.g. means) or other basic estimates (e.g. regression coefficient) AND variation (e.g. standard deviation) or associated estimates of uncertainty (e.g. confidence intervals)
☐	☒	For null hypothesis testing, the test statistic (e.g. *F*, *t*, *r*) with confidence intervals, effect sizes, degrees of freedom and *P* value noted<br>*Give P values as exact values whenever suitable.*
☒	☐	For Bayesian analysis, information on the choice of priors and Markov chain Monte Carlo settings
☒	☐	For hierarchical and complex designs, identification of the appropriate level for tests and full reporting of outcomes
☐	☒	Estimates of effect sizes (e.g. Cohen's *d*, Pearson's *r*), indicating how they were calculated

Our web collection on statistics for biologists contains articles on many of the points above.

Software and code

Policy information about availability of computer code

Data collection	REDCAP v13
Data analysis	R v2.4.0;<br>Plink v1.90b6.17 and v2.00a3LM

For manuscripts utilizing custom algorithms or software that are central to the research but not yet described in published literature, software must be made available to editors and reviewers. We strongly encourage code deposition in a community repository (e.g. GitHub). See the Nature Portfolio guidelines for submitting code & software for further information.

Data

Policy information about availability of data

All manuscripts must include a data availability statement. This statement should provide the following information, where applicable:

- Accession codes, unique identifiers, or web links for publicly available datasets
- A description of any restrictions on data availability
- For clinical datasets or third party data, please ensure that the statement adheres to our policy

The BioVU subject-level data are available under restricted access based on the requirements of the participant consent process. Access to BioVU clinical and genetic data is controlled by the BioVU data repository (https://victr.vumc.org/biovu-description/#). Upon publication, data sets of individual-level phenotype data

and corresponding data dictionaries to replicate the primary findings for the bone marrow biopsy outcomes, taxane and azathioprine studies presented here for research purposes will be made available upon request from the repository (biovu@vumc.org). BioVU vetting for use of individual-level data includes institutional IRB approval, data use agreements, and administrative and scientific reviews. eMERGE data are available through dbGaP (phs001584.v2.p2) and a list of deposited data and links can be found at https://emerge-network.org/dbgap/. Additional eMERGE phenotype data can be requested at https://emerge-network.org/contact/. The data generated in this study related to bone marrow biopsy outcomes, taxane treatment and azathioprine discontinuation are provided in the Supplementary Information/Source Data file.

Research involving human participants, their data, or biological material

Policy information about studies with human participants or human data. See also policy information about sex, gender (identity/presentation), and sexual orientation and race, ethnicity and racism.

Reporting on sex and gender	These analyses were performed on a genotyped population, and genetic sex was determined based on genotype. There were no pre-specified analyses that stratified by sex. For the studies presented, there were too few outcomes to perform an analysis stratified by sex.
Reporting on race, ethnicity, or other socially relevant groupings	The study populations were of European ancestry, as determined by genetic principal components analysis. There was no reporting on race, ethnicity of other socially relevant groupings.
Population characteristics	The manuscript contains multiple study populations that were derived from a genotyped population of 71078 participants. The sex breakdown of the cohort is 39595 females and 31483 males. The demographics of the individual study populations used to address specific research questions are presented in the manuscript.
Recruitment	There was no recruitment. These are analyses of existing data.
Ethics oversight	These studies were evaluated by the Vanderbilt University Medical Center Institutional Review Board (IRB) and determined to be non-human subjects research.

Note that full information on the approval of the study protocol must also be provided in the manuscript.

Field-specific reporting

Please select the one below that is the best fit for your research. If you are not sure, read the appropriate sections before making your selection.

☒ Life sciences ☐ Behavioural & social sciences ☐ Ecological, evolutionary & environmental sciences

For a reference copy of the document with all sections, see nature.com/documents/nr-reporting-summary-flat.pdf

Life sciences study design

All studies must disclose on these points even when the disclosure is negative.

Sample size	These were analyses of existing data, and sample size was determined by data availability.
Data exclusions	The primary exclusion was not being of European Ancestry. As detailed in the manuscript, these analyses examined outcomes and data where results for African Ancestry populations have been reported. These analyses examined whether the findings were relevant to European Ancestries.
Replication	Findings were replicated by (1) confirming associations were consistent across multiple clinical settings (frequency of ICD codes for a low WBC count, bone marrow biopsy outcomes, frequency of and ICD code drug-induced leukopenia and medication discontinuation rates) using distinct data sets in BioVU; and (2) examining the same ICD-code and drug-induced leukopenia outcomes in external data from other institutions to determine the replicability of the findings. All replication attempts are reported, and were successful in the external data sets.
Randomization	All analyses were adjusted for race, sex and genetic principal components. For analyses examining the outcomes of bone marrow biopsies, covariates that were clinically relevant to the outcome were included.
Blinding	In instances where manual data extraction was performed, the investigators were blinded to genotype (the exposure of interest) of the participants. For all other experiments, analyses were based on existing data derived from electronic health records. The investigators were not involved in the generation of the clinical data, so blinding was not relevant.

Reporting for specific materials, systems and methods

We require information from authors about some types of materials, experimental systems and methods used in many studies. Here, indicate whether each material, system or method listed is relevant to your study. If you are not sure if a list item applies to your research, read the appropriate section before selecting a response.

Materials & experimental systems

n/a	Involved in the study
☒	☐ Antibodies
☒	☐ Eukaryotic cell lines
☒	☐ Palaeontology and archaeology
☒	☐ Animals and other organisms
☒	☐ Clinical data
☒	☐ Dual use research of concern
☒	☐ Plants

Methods

n/a	Involved in the study
☒	☐ ChIP-seq
☒	☐ Flow cytometry
☒	☐ MRI-based neuroimaging

Plants

Seed stocks	*Report on the source of all seed stocks or other plant material used. If applicable, state the seed stock centre and catalogue number. If plant specimens were collected from the field, describe the collection location, date and sampling procedures.*
Novel plant genotypes	*Describe the methods by which all novel plant genotypes were produced. This includes those generated by transgenic approaches, gene editing, chemical/radiation-based mutagenesis and hybridization. For transgenic lines, describe the transformation method, the number of independent lines analyzed and the generation upon which experiments were performed. For gene-edited lines, describe the editor used, the endogenous sequence targeted for editing, the targeting guide RNA sequence (if applicable) and how the editor was applied.*
Authentication	*Describe any authentication procedures for each seed stock used or novel genotype generated. Describe any experiments used to assess the effect of a mutation and, where applicable, how potential secondary effects (e.g. second site T-DNA insertions, mosiacism, off-target gene editing) were examined.*
